# Cardiovascular magnetic resonance characterization of myocardial tissue injury in a miniature swine model of cancer therapy-related cardiovascular toxicity

**DOI:** 10.1016/j.jocmr.2024.101033

**Published:** 2024-03-07

**Authors:** Kei Nakata, Selcuk Kucukseymen, Xiaoying Cai, Tuyen Yankama, Jennifer Rodriguez, Eiryu Sai, Patrick Pierce, Long Ngo, Shiro Nakamori, Nadine Tung, Warren J. Manning, Reza Nezafat

**Affiliations:** a Department of Medicine, Cardiovascular Division, Beth Israel Deaconess Medical Center and Harvard Medical School, Boston, Massachusetts, USA; b Siemens Medical Solutions USA, Inc., Boston, Massachusetts, USA; c Department of Cardiology and Nephrology, Mie University Graduate School of Medicine, Tsu, Japan

**Keywords:** Anthracycline, Cancer therapy-related cardiac dysfunction, Cardiovascular magnetic resonance, Magnetic resonance spectroscopy, Miniature swine, Native T_1_ and T_2_ mapping

## Abstract

**Background:**

Left ventricular ejection fraction (LVEF) is the most commonly clinically used imaging parameter for assessing cancer therapy-related cardiac dysfunction (CTRCD). However, LVEF declines may occur late, after substantial injury. This study sought to investigate cardiovascular magnetic resonance (CMR) imaging markers of subclinical cardiac injury in a miniature swine model.

**Methods:**

Female Yucatan miniature swine (n = 14) received doxorubicin (2 mg/kg) every 3 weeks for 4 cycles. CMR, including cine, tissue characterization via T_1_ and T_2_ mapping, and late gadolinium enhancement (LGE) were performed on the same day as doxorubicin administration and 3 weeks after the final chemotherapy cycle. In addition, magnetic resonance spectroscopy (MRS) was performed during the 3 weeks after the final chemotherapy in 7 pigs. A single CMR and MRS exam were also performed in 3 Yucatan miniature swine that were age- and weight-matched to the final imaging exam of the doxorubicin-treated swine to serve as controls. CTRCD was defined as histological early morphologic changes, including cytoplasmic vacuolization and myofibrillar loss of myocytes, based on post-mortem analysis of humanely euthanized pigs after the final CMR exam.

**Results:**

Of 13 swine completing 5 serial CMR scans, 10 (77%) had histological evidence of CTRCD. Three animals had neither histological evidence nor changes in LVEF from baseline. No absolute LVEF <40% or LGE was observed. Native T_1_, extracellular volume (ECV), and T_2_ at 12 weeks were significantly higher in swine with CTRCD than those without CTRCD (1178 ms vs. 1134 ms, p = 0.002, 27.4% vs. 24.5%, p = 0.03, and 38.1 ms vs. 36.4 ms, p = 0.02, respectively). There were no significant changes in strain parameters. The temporal trajectories in native T_1_, ECV, and T_2_ in swine with CTRCD showed similar and statistically significant increases. At the same time, there were no differences in their temporal changes between those with and without CTRCD. MRS myocardial triglyceride content substantially differed among controls, swine with and without CTRCD (0.89%, 0.30%, 0.54%, respectively, analysis of variance, p = 0.01), and associated with the severity of histological findings and incidence of vacuolated cardiomyocytes.

**Conclusion:**

Serial CMR imaging alone has a limited ability to detect histologic CTRCD beyond LVEF. Integrating MRS myocardial triglyceride content may be useful for detection of early potential CTRCD.

## 1 Background

Anthracycline chemotherapy remains essential in treating many forms of cancer [1]. In the adjuvant setting for breast cancer, anthracycline-based chemotherapy dramatically improves recurrence-free and overall survival [2]. However, studies have demonstrated that 1.6%−2.1% of patients treated with anthracyclines develop early-onset cancer therapy-related cardiac dysfunction (CTRCD) during therapy or within the first year after treatment, and 1.6%−5% develop late-onset chronic CTRCD [3], [4]. Under current guidelines, transthoracic echocardiography, including 3D-left ventricular (LV) ejection fraction (LVEF) and LV global longitudinal strain (GLS) assessment, is the recommended imaging technique for monitoring CTRCD [4], [5], [6], [7], [8]. GLS is especially important in patients with low-normal LVEF to confirm asymptomatic myocardial injury [9]. A recent study demonstrated the utility of GLS in surveillance for CTRCD [10]. However, a limitation of GLS is its load dependency and reproducibility [11]. Patients receiving chemotherapies can experience blood pressure and intravascular volume changes, potentially impacting GLS measurements, even without evidence of myocardial injury. Also, GLS-guided management for CTRCD may not be superior to an LVEF-guided management [12]. Therefore, there are ongoing controversies about the utility of GLS for guiding treatment [13].

Cardiovascular magnetic resonance (CMR) enables an accurate and reproducible measure of function, strain, tissue composition, and metabolism. Theoretically, cellular damage at the myocardial tissue level occurs before LV systolic dysfunction and heart failure [14], and may be identified by CMR tissue characterization. Mitochondrial dysfunction memory has been considered a critical mechanism in anthracycline-based cumulative and progressive development of heart failure [15], [16]. Myocardial lipid metabolism is largely dependent on the mitochondria to generate energy in cells [17]. ^1^H-magnetic resonance (MR) spectroscopy (MRS) can measure in-vivo myocardial triglyceride (TG) content and potentially detect metabolic changes due to anthracycline treatment [18]. Some studies have reported elevated baseline native T_1_ and increased native T_1_ during chemotherapy as an important predictor for CTRCD [19], [20] in patients undergoing anthracycline-based chemotherapy, while other studies have demonstrated these CMR tissue biomarkers are not useful for assessing cardiovascular toxic effects that may result in CTRCD. This highlights current controversies in the potential utility of CMR for detecting early CTRCD [21], [22].

CMR in small or large animal models of CTRCD with histological validation could provide more precise evidence of the potential of CMR to detect myocardial tissue damage. Myocardial native T_1_ and T_2_ have been used to detect early chemotherapy-related changes of myocardial tissue in mouse [23] and rabbit models [24], [25], suggesting that native T_1_ and T_2_ have the potential to detect early CTRCD. In a large animal study with *intracoronary* anthracycline injection by Galán-Arriola et al., T_2_ mapping (but not T_1_ and extracellular volume [ECV] mapping), identified intracardiac edema as the earliest marker of CTRCD [26]. In a Yorkshire swine model receiving *intravenous* anthracycline injection, we have also recently reported that serial myocardial T_1_ and T_2_ mapping during anthracycline treatment can potentially assess for early anthracycline-related CTRCD beyond LVEF assessment [27]. Studies using metabolomic profiling with MRS of in-vitro samples have also shown metabolic changes in a rat model [28]. While these pre-clinical studies demonstrate the potential utility of CMR in detecting subclinical CTRCD, we acknowledge limitations. Intravenous infusion or high-dose anthracycline may cause significant tissue damage, not necessarily recapitulating human CTRCD. In immature large animal models where the heart may still be growing, myocardial tissue changes due to increased cardiomyocyte number (hyperplasia) or enlargement of individual cardiomyocytes (hypertrophy) may be a confounding issue [29].

In this study, we sought to investigate the role of serial CMR and serum biomarkers for detecting the early-stage CTRCD, as determined by histological assessment, using miniature swine receiving anthracycline doses similar to the current dosing regimen in breast cancer patients. Furthermore, in a pilot cross-sectional study, the potential of MRS to detect underlying myocardial tissue damage was investigated in a subset of animals.

## 2 Methods

### 2.1 Animal model

Miniature swine provide advantages in long-term CRTCD studies in terms of slow and limited growth, even at full maturity. The size of the heart and blood vessels is more analogous to that in humans than in swine, dogs, or nonhuman primates, so it may help better understand the potential effects of anthracyclines on the myocardium. Seventeen female-gender Yucatan miniature swine were studied, 14 of which [6–7 months old, 29–38 kg] received serial CMR and chemotherapy over 12 weeks, and 3 control swine were fed on farms and underwent CMR at 12 weeks [10–11 months old, 46–58 kg]. Due to logistical and cost issues, we sought to match the age/weight of the control animals to those under therapy at 12 weeks (i.e., 6/7 months + 4 months). The protocol was approved by the Institutional Animal Care and Use Committee and conformed to the position of the American Heart Association on Research Animal Use and the Declaration of Helsinki. Before the planned experiment day, all animals had 5–7 days to allow adjustment and acclimation to the indoor animal facility.

## 3 Study protocol

**Fig. 1:**
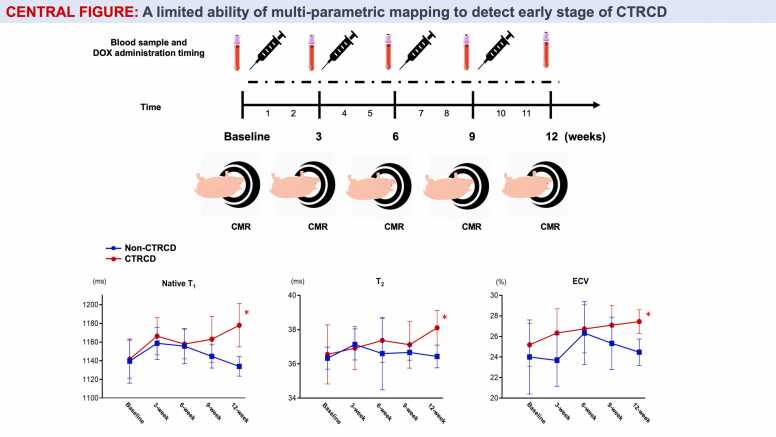
Experimental timeline and numbers of animals euthanized for histological assessment. All swine receiving chemotherapy received serial CMR scans followed by DOX injection (2.0 mg/kg) every 3 weeks (Group 1: without MRS, Group 2: with MRS at 12 weeks). One swine was euthanized immediately after 4th injection secondary to severe bleeding. Extracted hearts from the remaining 13 DOX-treated swine were submitted for pathological assessment. Three swine served as controls. They underwent CMR with MRS and were euthanized for histological assessment at 12 weeks. CMR: cardiovascular magnetic resonance, DOX: doxorubicin, MRS: magnetic resonance spectroscopy.

Fig. 1 shows the study protocol and experimental timeline. In the chemotherapy group, all swine received sequential CMR scans followed by same-day doxorubicin (DOX) injection (2.0 mg/kg) every 3 weeks [27], a dose of regimen similar to adjuvant chemotherapy for human breast cancer (60 mg/m^2^, every 3 weeks). The swine completed 4-cycle injections and were euthanized 3 weeks after the 4th cycle. Before each CMR/DOX procedure, general anesthesia was performed. After 12 h of fasting, sedation was initiated with a 1.4 mg/kg intramuscular injection of tiletamine/zolazepam hydrochloride (Telazol; Fort Dodge Animal Health, Fort Dodge, Iowa, USA). A percutaneous venous ear catheter was placed, followed by a 1–3 mL blood sample collection. Endotracheal intubation was performed, and general anesthesia was maintained with isoflurane inhalation (2%–4%). Ventilation was maintained between 10 and 16 breaths/min with tidal volumes between 200 and 350 mL. Animals were monitored and transported to the CMR room, and 5 mg of metoprolol was administered before CMR scanning. Heart rate and electrocardiography (ECG) data were monitored using a 3-lead ECG device and recorded every 15 min. A12-lead ECG was recorded before DOX injection with a paper speed of 25 cm/s and an amplification of 10 mm/mV. Control female swine weighing 46–58 kg were fed in the farm and euthanized with histology after 12-week CMR scan.

### 3.1 CMR image acquisition and data analysis

**Fig. 2:**
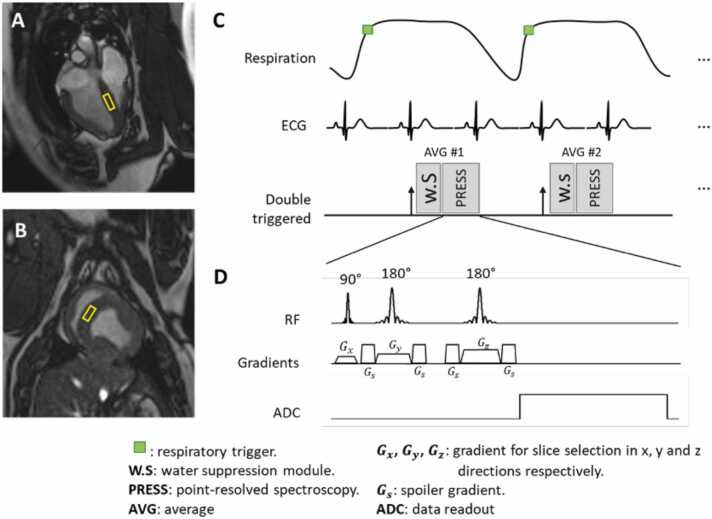
MR spectroscopy. (A, B) Positioning of single voxel on the septum in 4-chamber and short-axis planes. (C) Double triggering scheme for motion control. The excitations in point-resolved spectroscopy (PRESS) are triggered to end-expiration with diaphragm-based navigator and to end-systole with ECG signal. (D) PRESS sequence diagram. The 3 RF pulses, which are slice selective in 3 orthogonal directions, and gradients are shown. For acquisition without water suppression (W.S.), the W.S pulses are replaced with additional trigger delay of equivalent duration. AVG, average; ECG, electrocardiogram; PRESS, point-resolved spectroscopy; RF, radiofrequency; W.S., water suppression; ADC, analog digital converter.

CMR images were acquired using a 3T scanner (MAGNETOM Vida, Siemens Healthineers, Erlangen, Germany) with an 18-channel phased-array coil. To assess LV systolic function and volume, 10–12 short-axis stack images and 2- and 4-chamber long-axis images were acquired using a cine balanced steady-state free precession sequence (slice thickness, 8 mm; gap, 2 mm, in-plane spatial resolution 1.6 × 1.6 mm) [30]. Pre- and post-contrast T_1_ mapping were obtained from 3 short-axis images (basal, mid-ventricular, and apical) of the LV using a modified look-locker inversion recovery sequence based on a 5(3)3 scheme with the following parameters: diastolic acquisition, 3 slices, T_1_ mapping; repetition time/echo time = 2.5/1.0 ms, flip angle = 35°, field of view (FOV) = 360 × 325 mm^2^, voxel size = 1.7 × 1.7 mm^2^, slice thickness = 8 mm. T_2_ mapping was acquired in the same locations as the T_1_ maps using T_2_ preparation pulses and gradient echo readout with the following parameters: repetition time/echo time = 2.5/1.3 ms, flip angle = 12°, FOV = 360 × 325 mm^2^, voxel size = 1.9 × 1.9 mm^2^, slice thickness = 8 mm, No. of T_2_ preps = 3 (duration 0, 30, 55 ms). 3D late gadolinium enhancement (LGE) images were acquired using an inversion recovery sequence 10–20 min after contrast injection of 0.2 mmol/kg of gadobutrol (Gadavist, Bayer Healthcare, Berlin, Germany) with the following imaging parameters: TE = 1.3 ms, flip angle = 55°, FOV = 350 × 350 × 125 mm^3^, acquisition matrix = 224 × 224, spatial resolution = 1.6 × 1.6 × 4 mm^3^ to 6 mm. The ^1^H-MRS data were collected at 12 weeks in 10 swine using point-resolved spectroscopy (PRESS) [31] on a septal voxel (Fig. 2) during free-breathing using the following parameters: voxel size = 20 × 20 × 10 mm^3^, TE = 33 ms, 32 averages, 512 samples with 2500 Hz bandwidth, effective repetition time > 2500 ms, respiratory triggered to end-expiration and ECG-triggered to end-systole. Each spectrum took about 3.75 min to acquire. Two spectra were acquired, with and without water suppression, respectively.

CMR data were analyzed using CVI42 workstation (v. 5.11.1, Circle Cardiovascular Imaging, Inc., Calgary, Alberta, Canada). Epi- and endocardial contours of the LV and right ventricle (RV) were generated in contiguous short-axis cine images using the automatic contour function, then manually adjusted if required. LV papillary muscle mass was isolated and added to LV mass. RV papillary muscles and trabeculations were included in the RV volume. For feature-tracking analyses, GLS was assessed in 3 LV long-axis views: 4-ch, 3-ch, and 2-ch. Global radial and circumferential strain was assessed in short-axis stack (LV full coverage). RV free wall longitudinal strain (RV FWLS) was determined from the long-axis 4-chamber view. Left atrial (LA) strain was assessed from the long-axis 2-ch and 4-ch cine images, excluding the LA appendage and pulmonary veins. Epicardial and endocardial contours were generated using the automatic contour function, then manually adjusted for correct tracking if required. GLS and LA strains were calculated as the peak of the mean of the separate long axes. Myocardial native T_1_ and T_2_ were measured by placing a relatively large region of interest of size in the mid-ventricular septal myocardium on generated maps. T_1_ values of LV myocardium and blood pool were similarly determined before and after contrast injection, and ECV was computed as previously described [32]. The presence and pattern of non-ischemic LGE lesions were qualitatively assessed by consensus agreement of 2 experienced observers. The MRS sequence was implemented later in the course of the study, and myocardial TG content was measured at 12 weeks by MRS in 7 DOX-treated swine and 3 control swine. All ^1^H-MRS spectra were fitted using the on-scanner software (syngo.MR Spectro, Siemens Healthineers) with a time-domain fitting algorithm. Water-suppressed spectra were fitted to estimate myocardial TG peaks at 0.9, 1.3, and 2.1 ppm. Non-water-suppressed spectra were used to fit the water peak at 4.7 ppm. Myocardial TG content was calculated as the ratio of the area under TG peaks at 0.9 and 1.3 ppm to the area under the water peak.

### 3.2 Histological assessment

Three weeks after the completion of the 4th DOX injection and after their final (5th) CMR exam, swine were euthanized. The hearts were immediately harvested and fixed in 10% buffered formalin. After tissue fixation, the hearts were serially sectioned parallel to the atrioventricular groove into 5-mm-thick slices. The tissue samples corresponding to the basal, mid-ventricular, and apical slices on CMR T_1_ and T_2_ mapping were embedded in paraffin wax, sectioned at 5-μm thickness, and stained with Masson-trichrome to determine histological collagen volume fraction or with hematoxylin-eosin for analysis of the extra-cellular space component. The frequency and severity of DOX-induced cardiac lesions, including myofibrillar loss and cytoplasmic vacuolation, were assessed on the hematoxylin-eosin-stained samples by light microscopic examination and scored using morphologic grading systems [33], [34]. CTRCD was diagnosed based on histopathological findings (≥Grade 1) (see Table 1). Histological extracellular space component or collagen volume fraction was also quantified. Ten high-power (×200) magnification digital images from each myocardial segment (160 images per animal) were acquired for semi-automated image analysis (BZ-X700; Keyence, Osaka, Japan). Colored images with Masson-trichrome staining were split into red, green, and blue channels. Using the image with the best separation of myocardial fibrosis, the collagen area was derived from a combination of standard deviation (SD) from mean signal and isodata 16 automatic thresholding, and collagen volume fraction was calculated as a percentage of collagen area divided by total myocardial area. The image processing with a freehand selection tool was performed to exclude subendocardial or perivascular areas. The average quantification of the different images was considered the final value of fibrosis for the segment and animal. Similarly, in a hematoxylin-eosin stained section, a color-based calculation algorithm derived from bright pink staining of myocyte and stained purple of nucleus was applied to yield percentage areas of extra-cellular space component [35] (Image-J, version 1.50b; National Institutes of Health, Bethesda, Maryland).

**Table 1:** Histological data.

	Controls (n = 3)	CTRCD (n = 10)	Non-CTRCD (n = 3)
Billingham scoring system			
Grade 0 (Normal)	2 (67)	0	1 (33)
Grade 0.5 (Not completely normal)	1 (33)	0	2 (67)
Grade 1 (Minimal involvement of <5%)	0	4 (40)	0
Grade 1.5 (Involvement of <15%)	0	2 (20)	0
Grade 2 (Involvement of ≤25% and/or vacuolization)	0	4 (40)	0
Grade 2.5 (Involvement of ≤35% and/or vacuolization)	0	0	0
Grade 3 (Involvement of >35%)	0	0	0
Histological extra-cellular space	14.6 ± 1.8	16.0 ± 2.0	15.0 ± 4.0
Histological collagen volume fraction	3.4 ± 0.7	7.7 ± 3.1	4.4 ± 0.5

*CTRCD* cancer therapy-related cardiac dysfunction.

Grade 0 Normal myocardial ultrastructural morphology.

Grade 0.5 Not completely normal but no evidence of anthracycline-specific damage.

Grade 1 Isolated myocytes affected and/or early myofibrillar loss; damage to <5% of all cells.

Grade 1.5 Changes similar to those in grade 1 except damage involves 6%–15% of all cells.

Grade 2 Clusters of myocytes affected by microfibrillar loss and/or vacuolization, with damage 16%–25% of all cells.

Grade 2.5 Many myocytes (26%–35% of all cells) affected by vacuolization and/or myofibrillar loss.

Grade 3 Severe, diffuse myocyte damage (>35% of all cells).

Data are numbers (%) of cases or means ± standard deviation

### 3.3 Blood sampling and analysis

Blood samples were allowed to clot at room temperature for 30 min, centrifuged at 3000 rotation per minute for 15 min, aliquoted, and frozen for storage at −20°C. We measured serum cardiac troponin I (TnI) by homogeneous, sandwich chemiluminescent immunoassay based on LOCI technology (Siemens Healthcare) and measured N-terminal pro-B-type natriuretic peptide (NT-pro BNP) using a 1-step sandwich chemiluminescent immunoassay also based on LOCI technology. Both cardiac TnI and NT-pro BNP were measured on the Siemens Dimension EXL200 integrated analyzer at the Department of Comparative Medicine, Stanford University, Stanford, California.

### 3.4 Statistical analysis

Continuous variables are expressed as mean ± SD and compared using an unpaired Student's t-test or Mann-Whitney nonparametric test if not normally distributed. Categorical variables were reported as counts and percentages and compared using a chi-square test. One-way analysis of variance with Bonferroni adjustment for multiple comparisons was applied following the 3 group comparisons. Data analysis with repeated measures data, such as the analyses of serial changes of parameters, were carried out using linear mixed-effects models where the within-subject correlated measurements were modeled using compound-symmetry variance-covariance structure. Depending on data distribution, a Pearson or Spearman correlation coefficient was calculated to investigate possible associations of continuous outcome measures. All tests were 2-sided, and a p-value <0.05 was considered statistically significant. All analyses were performed using SPSS (version 25.0, International Business Machines Inc., Armonk, New York) and MedCalc for Windows (version 14.8.1, MedCalc Software, Ostend, Belgium).

## 4 Results

### 4.1 Swine characteristics

Of 14 swine receiving chemotherapy, 1 swine (7%) died due to uncontrollable bleeding after the 4th anthracycline injection and before the 12-week CMR scan. Diarrhea was the most obvious overt side effect but controllable in all Yucatan miniature swine. During the experiment, weight increases occurred (1.2 ± 0.3 kg/week). Swine in the anthracycline chemotherapy and control groups had similar weights before euthanization (47.1 ± 5.2 kg and 53.5 ± 6.5 kg, p = 0.24). No significant changes in the 12-lead ECG occurred throughout the experiment in all swine.

Histological findings are summarized in Table 1. Cardiomyocyte vacuolation or myofiber cytoplasmic vacuolation (Grade 2) was observed in 4 (31%) swine. The mean histologic collagen volume fraction in the CTRCD group and the non-CTRCD group were 7.7 ± 3.1% and 4.4 ± 0.5%, and the extra-cellular space component in those was 16.0 ± 2.0% and 15.0 ± 4.0%, respectively. There was a significant difference in collagen volume fraction among the groups (analysis of variance, p = 0.04), and the CTRCD group had a higher collagen volume fraction than the control and the non-CTRCD groups (p = 0.02 and 0.08). Table 2 summarizes serum biomarkers and CMR parameters at 12 weeks of swine receiving anthracycline and controls. The hemoglobin level after each injection gradually decreased, and its level at 12 weeks was significantly lower in swine receiving anthracycline compared with controls. Myocardial native T_1_, T_2_, and ECV increased in the anthracycline-treated swine compared to controls, and were significantly higher in those with CTRCD than those without CTRCD. Cardiac TnI, NT-pro BNP, and strain parameters, such as LV GLS and RV FWLS, were also elevated in anthracycline-treated swine; however, there were no significant differences between the CTRCD and non-CTRCD groups. No LGE was observed in any of the animals. Absolute LVEF did not drop to <40% in any swine receiving chemotherapy.

**Table 2:** Comparisons of 12-week CMR data among swine with and without CTRCD, and controls.

	Controls (n = 3)	DOX treatment (n = 13)	p-Value Controls vs. DOX treatment	CTRCD (n = 10)	Non-CTRCD (n = 3)	p-Value CTRCD vs. non-CTRCD
BW, kg	53.5 ± 6.5	47.1 ± 5.2	0.24	47.3 ± 6.0	46.4 ± 2.0	0.68
WBC, 10^3^/µL	7.8 ± 0.4	7.7 ± 2.6	0.95	7.7 ± 2.3	7.7 ± 4.3	0.98
Hb, g/dL	12.6 ± 1.8	9.8 ± 1.3	0.02	10.0 ± 1.2	8.9 ± 1.1	0.20
HCT, %	34.1 ± 1.2	34.7 ± 3.4	0.63	33.9 ± 3.4	37.3 ± 1.8	0.13
TnI, ng/mL	0.003 ± 0.006	0.019 ± 0.011	0.01	0.021 ± 0.012	0.013 ± 0.006	0.26
CRP, mg/L	0.97 ± 0.55	1.15 ± 0.82	0.67	1.30 ± 0.87	0.63 ± 0.21	0.20
NT pro-BNP, pg/mL	0	0.34 ± 0.40	0.10	0.40 ± 0.43	0.13 ± 0.16	0.48
LVEDV, mL	83.5 ± 18.6	74.2 ± 12.0	0.48	75.7 ± 13.0	69.4 ± 7.7	0.34
LVEDVI, mL/m^2^	72.2 ± 10.8	70.2 ± 7.7	0.78	71.3 ± 7.8	66.5 ± 7.6	0.41
LVESV, mL	38.8 ± 9.7	36.2 ± 6.5	0.69	37.3 ± 6.7	32.2 ± 4.7	0.20
LVESVI, mL/m^2^	33.5 ± 6.0	34.2 ± 5.0	0.85	35.2 ± 4.8	30.9 ± 5.0	0.27
LVSV, mL	44.7 ± 8.9	37.9 ± 6.6	0.31	38.1 ± 7.4	37.1 ± 4.0	0.77
LVEF, %	53.8 ± 1.5	51.2 ± 4.1	0.23	50.4 ± 4.2	53.6 ± 2.9	0.19
LVM, g	63.5 ± 9.9	66.6 ± 9.7	0.66	68.0 ± 10.5	62.0 ± 4.7	0.20
LVMI, g/m^2^	55.1 ± 4.2	63.3 ± 8.0	0.14	64.5 ± 8.7	59.4 ± 3.0	0.15
Presence of LGE, n	0	0	-	0	0	-
Septum native T_1_, ms	1134 ± 8	1168 ± 28	0.009	1178 ± 23	1134 ± 10	0.002
Septum T_2_, ms	36.3 ± 0.7	37.7 ± 1.2	0.03	38.1 ± 1.0	36.4 ± 0.7	0.02
Septum ECV, %	24.3 ± 1.5	26.6 ± 1.8	0.10	27.4 ± 1.1	24.5 ± 1.3	0.03
2D LVGRS, %	29.3 ± 3.4	26.4 ± 3.6	0.28	26.1 ± 4.0	27.5 ± 0.8	0.36
2D LVGCS, %	−16.8 ± 1.7	-16.3 ± 1.6	0.70	−16.2 ± 1.8	−16.5 ± 0.8	0.70
2D LVGLS, %	−15.1 ± 1.5	-12.8 ± 2.6	0.08	−12.3 ± 2.6	−14.5 ± 2.4	0.25
PDRSR, 1/s	−2.7 ± 0.7	-2.8 ± 0.4	0.86	−2.6 ± 0.3	−3.3 ± 0.1	0.01
PDCSR, 1/s	1.7 ± 0.4	1.7 ± 0.2	0.93	1.7 ± 0.2	1.9 ± 0.2	0.07
PDLSR,1/s	1.5 ± 0.4	1.3 ± 0.5	0.53	1.2 ± 0.4	1.8 ± 0.2	0.05
RVEDV, mL	62.1 ± 9.5	61.5 ± 12.9	0.93	61.1 ± 14.0	62.8 ± 11.2	0.84
RVEDVI, mL/m^2^	53.9 ± 4.0	58.1 ± 9.3	0.26	57.4 ± 8.8	60.4 ± 12.4	0.73
RVESV, mL	24.4 ± 3.4	28.3 ± 7.5	0.21	28.7 ± 7.9	27.0 ± 6.9	0.74
RVESVI, mL/m^2^	21.2 ± 1.6	26.7 ± 5.6	0.06	26.8 ± 5.5	26.0 ± 7.3	0.87
RVEF, %	60.6 ± 3.0	54.3 ± 4.8	0.04	53.4 ± 5.0	57.3 ± 3.3	0.17
RV FWLS, %	−27.2 ± 2.6	-21.7 ± 4.7	0.06	−21.5 ± 4.7	−22.7 ± 5.2	0.74
LAV, mL	30.3 ± 5.2	28.2 ± 5.3	0.58	28.8 ± 5.8	26.4 ± 3.2	0.41
LAVI, mL/m^2^	26.3 ± 2.7	26.8 ± 4.6	0.81	27.2 ± 4.9	25.4 ± 3.8	0.54
LA strain, %	41.8 ± 2.4	32.8 ± 8.6	0.06	32.9 ± 8.3	32.5 ± 11.5	0.95
Myocardial TG, %*	0.89 ± 0.32	0.37 ± 0.15	0.04	0.30 ± 0.06	0.54 ± 0.18	0.05

*BW* body weight, *CRP* C-reactive protein, *CTRCD* cancer therapy-related cardiac dysfunction, *DOX* doxorubicin, *ECV* extracellular volume, *GCS* global circumferential strain, *GLS* global longitudinal strain, *GRS* global radial strain, *Hb* hemoglobin, *LA* left atrium, *LAV/LAVI* left atrial volume/left atrial volume index, *LGE* late gadolinium enhancement, *LVEDV/LVESV* left ventricular end-diastolic/systolic volume, *LVEDVI/LVESVI* left ventricular end-diastolic/systolic volume index, *LVEF* left ventricular ejection fraction, *LVM/LVMI* left ventricular mass/left ventricular mass index, *LVSV* left ventricular stroke volume, *NT-pro BNP* N-terminal pro brain natriuretic peptide, *PDCSR* peak diastolic circumferential strain rate, *PDLSR* peak diastolic longitudinal strain rate, *PDRSR* peak diastolic radial strain rate, *PLT* platelet, *RVEDV/RVESV* right ventricular end-diastolic/systolic volume, *RVEDVI/RVESVI* right ventricular end-diastolic/systolic volume index, *RVEF* right ventricular ejection fraction, *RV FWLS* right ventricular free wall longitudinal strain, *TG* triglyceride, *TnI* Troponin I, *WBC* white blood cell count.

Data are numbers (%) of cases or means ± standard deviation

* Ten swine (3 controls, 2 non-CTRCD, and 5 CTRCD) underwent myocardial TG assessment.

### 4.2 Serial changes of CMR parameters between CTRCD and non-CTRCD

**Fig. 3:**
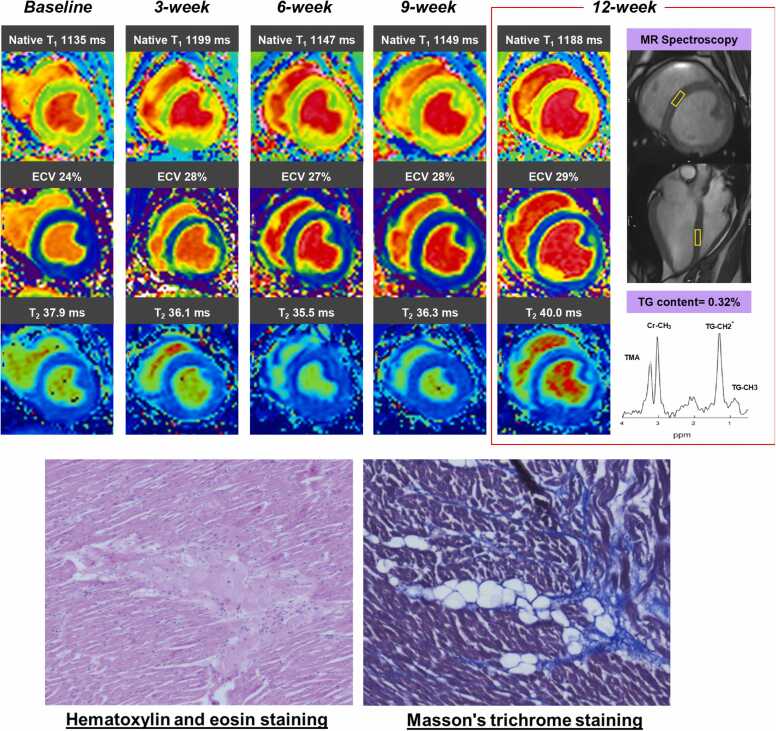
A representative case of CTRCD. Myocardial native T_1_ and ECV elevated immediately after the first injection, and slightly decreased at 6-week follow-up. The increases in native myocardial T_1_ and ECV were more pronounced at 12-week follow-up. Serial changes of native myocardial T_1_ and ECV were quite similar. Meanwhile, T_2_ was not changed until 9-week follow-up, and significantly elevated at 12-week follow-up. Histological samples at 12 weeks showed severe myocardial injury, including myocyte swelling and vacuolated myocyte with reactive interstitial fibrosis, suggesting early phase of CTRCD. In this case, myocardial TG content was also decreased compared with the controls. CTRCD: cancer therapy-related cardiac dysfunction, ECV: extracellular volume, TG: triglycerides.

**Fig. 4:**
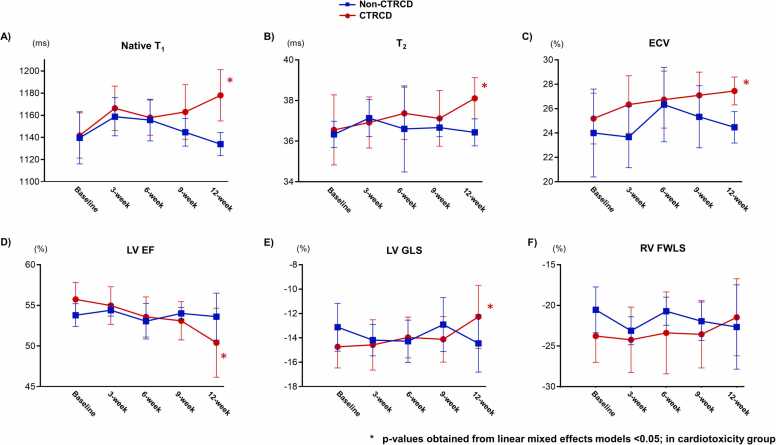
Serial native myocardial native T_1_, T_2,_ ECV, LVEF, LV GLS, and RV FWLS between CTRCD and non-CTRCD groups. (A, B, C) The temporal trends in native T_1_, T_2_, and ECV in the CTRCD group showed similar and significant rises, although there was overlap and heterogeneity between and within the groups. (D, E) This trend was also observed in LVEF and LV GLS with substantial overlap. (F) There was no temporal trend of RV FWLS in the CTRCD group with significant overlap at the individual animal level. CTRCD: cancer therapy-related cardiac dysfunction, ECV: extracellular volume fraction, LVEF: left ventricular ejection fraction, LV GLS: left ventricular global longitudinal strain, RV FWLS: right ventricular free wall longitudinal strain.

Fig. 3 depicts serial CMR images and histology from a case with CTRCD with LVEF ≥50%. Fig. 4 shows the mean ± SD of CMR parameters during experiments in swine with and without CTRCD. In swine with CTRCD, native T_1_, T_2_, ECV, LVEF, LV GLS, and RV FWLS at baseline were 1142 ms, 36.6 ms, 25%, 55.7%, −14.7%, and −23.8%, respectively, which were similar with those in swine without CTRCD (1140 ms, 36.3 ms, 24%, 53.8%, −13.1%, and −20.5%, respectively, all p values >0.05). Native T_1_, T_2_, and ECV at 12 weeks were significantly higher in swine with CTRCD, and their temporal increases in the CTRCD group were statistically significant (native T_1_: 1142 ms [baseline] to 1178 ms [12-week], p = 0.003, T2: 36.6 ms [baseline] to 38.1 ms [12-week], p = 0.001, ECV: 25% [baseline] to 27% [12-week], p = 0.01, respectively). However, there were no differences in temporal trends of native T_1_, ECV, and T_2_ between the groups with and without CTRCD (p = 0.05, 0.08, and 0.22, respectively). The temporal changes in LVEF and LV GLS also differed significantly in the CTRCD group (LVEF: 55.7% [baseline] to 50.4% [12-week], p < 0.001, LV GLS: −14.7% [baseline] to −12.3% [12-week], p = 0.009, respectively); however, no statistical significance between groups was observed due to considerable temporal variability and overlap (p = 0.9 and 0.8, respectively). Also, there were no temporal trends in RV FWLS, cardiac TnI, NT-pro BNP, and C-reactive protein levels in the CTRCD group.

### 4.3 Myocardial TG content using ^1^H-MRS

**Fig. 5:**
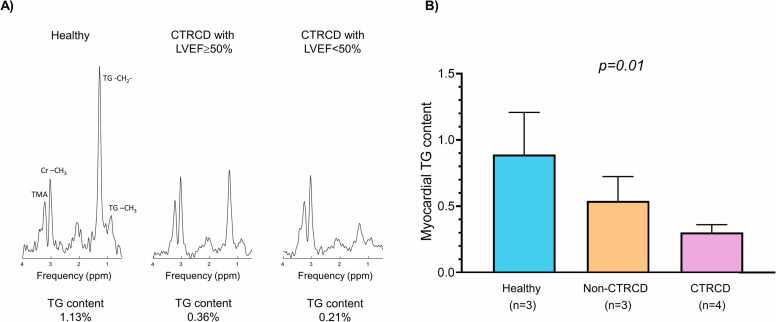
Exemplary ^1^H-MRS and comparison of myocardial TG content. (A) A case from control group and cases with CTRCD. Multiple metabolite resonance peaks are shown, including the TG peaks at 0.9 and 1.3 ppm, creatine at 3.0 ppm, and TMA at 3.2 ppm. The TG peaks (TG -CH_2_^−^ and -CH_3_) in CTRCD cases have smaller magnitudes compared to those in the control. Myocardial TG content of healthy, CTRCD without LV systolic dysfunction, and CTRCD with LV systolic dysfunction are 1.13%, 0.36%, and 0.21%, respectively. (B) Myocardial TG content in swine with anthracycline chemotherapy was significantly lower compared with a control (p = 0.006). There was a statistical difference in myocardial TG among the 3 groups (p = 0.01). CTRCD: cancer therapy-related cardiac dysfunction, LVEF: left ventricular ejection fraction, MRS: magnetic resonance spectroscopy, TG: triglycerides, TMA: trimethylamines.

Fig. 5A illustrates exemplary MRS spectra obtained from 1 healthy control (A, myocardial TG = 1.13%) and 2 anthracycline-treated swine (B, myocardial TG = 0.36%; C, myocardial TG = 0.21%). The TG peaks at 0.9 and 1.3 ppm of the anthracycline-treated swine had lower magnitudes than those of the controls. There was a statistical difference in myocardial TG among healthy controls, non-CTRCD, and CTRCD groups (p = 0.04). TG content in anthracycline-treated swine was significantly lower compared with controls (0.37 ± 0.15% and 0.89 ± 0.32%, p = 0.006) and appeared to be lower in swine with CTRCD than those without CTRCD (0.30 ± 0.06% and 0.54 ± 0.18%, p = 0.05) Fig. 5B. TG content assessed by ^1^H-MRS was strongly associated with the incidence of cardiac vacuolization (p = 0.03) and histological collagen volume fraction (r = −0.70, p = 0.02). In addition, TG content appeared to be correlated with LV GLS (r = −0.68, p = 0.03) and myocardial native T_1_ (r = −0.53, p = 0.11).

## 5 Discussion

In this pre-clinical study of anthracycline-induced CTRCD mimicking human clinical breast cancer protocols, we found that 1) there was a significant difference in myocardial native T_1_, T_2_, and ECV values at 12-week follow-up between CTRCD and non-CTRCD groups; 2) the temporal trends in native T_1_, T_2_, ECV in CTRCD group showed statistically significant increases although there was overlap and heterogeneity between and within the groups. This trend was also observed in LVEF and LV GLS with substantial overlap; 3) myocardial TG assessed by ^1^H-MRS at 12 weeks was markedly decreased in anthracycline-treated swine and highly associated with the severity of histological myocardial damages.

A key preliminary finding of our study is that after 4-cycle anthracycline treatment, myocardial TG content was markedly reduced and levels correlated with histological myocardial damage. The role of apoptosis in anthracycline-induced CTRCD is well established; however, in recent years, emerging regulated cell death pathways, such as ferroptosis, have been implicated in CTRCD [36]. Ferroptosis is a type of programmed cell death dependent on iron and characterized by the accumulation of lipid peroxides. The exaggerated lipid peroxidation and excess iron due to anthracycline treatment may contribute to reducing the cardiomyocyte TG pool needed to provide substrates for fatty acid oxidation and maintaining myocardial energy balance. Although myocardial TG was recognized as a site of ectopic fat accumulation, it seems to play a potential role in myocardial energy metabolism for the development of CTRCD. Indeed, TG content was significantly decreased after anthracycline injection even without histological evidence of CTRCD, suggesting the potential of myocardial TG suppression to detect early potential CTRCD. Furthermore, myocardial TG suppression was enhanced in swine with CTRCD and associated with potential sequelae, such as vacuolization and fibrosis after myocyte cell death. The preliminary data suggest the potential role of ^1^H-MRS myocardial TG content for predicting CTRCD. These findings should be considered preliminary and should be validated in larger studies. Understanding the mechanisms by which anthracycline leads to cardiomyocyte death may lead to more targeted approaches to cardiotoxicity testing.

Population-based studies described early myocardial native T_1_, T_2_, and ECV changes as potential biomarkers for detecting CTRCD [37], [38], [39]. We also found a difference in myocardial native T_1_, T_2_, and ECV values at 12-week follow-up between CTRCD and non-CTRCD groups with substantial variability between individual animals. Nevertheless, these differences are small and would be unlikely to reflect any definite differential changes in an individual patient, albeit with the need to individualize both interruptions or discontinuations of cancer therapy and cardioprotective drug initiation for patient-centered care and shared decision-making in cardio-oncology. In addition, it is unclear what the clinical significance of small tissue parameter increases would be and warrants further studies.

The present study failed to reach a statistical significance in the between-group temporal change of tissue parameters, with the difference in native T_1_, T_2_, and ECV from baseline to 12 weeks being 36 ms, 1.6 ms, 2.3% in the CTRCD group vs −6 ms, 0.1 ms, 0.1% in the non-CTRCD group (each p-value; 0.05, 0.08, and 0.22, respectively). Lower rates of CTRCD may result in even smaller differences and thus require larger samples in a potential experiment scenario. In a preclinical animal study, CTRCD developed through the preferential iron accumulation inside the mitochondria following anthracycline treatment [40]. Studies have reported that anthracycline-induced CTRCD is associated with iron overload and that iron potentiates anthracycline-induced oxidative stress [41], [42], indicating the coexistence of apoptosis, myocardial edema, fibrosis, and iron accumulation in the acute phase of CTRCD. Therefore, one potential source could be attributed to the confounding effect of intra-myocardial iron accumulation, which typically reduces native T_1_ and T_2_, on increased native T_1_ and T_2_ secondary to apoptosis, myocardial edema, and fibrosis. However, these findings could be critical in detecting early CTRCD and be an important consideration that would hamper the usability of multiparametric mapping for predicting early potential CTRCD. Altaha et al. validated quantitative CMR tissue characterization to identify CTRCD in patients receiving anthracycline and trastuzumab therapy using a 1.5T CMR scanner at baseline, 3, and 6-month intervals [21]. In a study by Thavendiranathan et al., the increases in native T_1_, T_2_, and ECV during chemotherapy were transient and were not useful in identifying traditional CTRCD risk [22]. Given these considerations, the current multiparametric mapping approach alone does not seem to have the necessary sensitivity needed to detect early anthracycline-related CTRCD in clinical practice and might not have a role in the routine surveillance of patients treated with potentially cardiotoxic cancer therapy. Integrating myocardial TG to multiparametric mapping likely reflects complementary information obtained from the different underlying pathophysiological processes of CTRCD. Subsequent linkage to relevant metabolic profiling, such as myocardial TG, may establish more sophisticated multiparametric CMR models for detecting early potential CTRCD.

## 6 Limitations

Small sample sizes and inherent variability between individual animals are limitations of our study. The follow-up time of 3 months was limited, and our study does not elucidate the long-term impact and the potential relationship between chemotherapy and radiotherapy, a cornerstone of oncologic treatment for breast cancer. We only had ^1^H-MRS data in a subset of animals due to technical challenges in implementing a free-breathing MRS sequence in our scanner. Therefore, temporal changes in myocardial TG during chemotherapy were not assessed. Prolonged fasting of more than 48 h can influence myocardial TG content measurement. However, no swine had more than 12 h of fasting in this study. No immunohistochemical staining was performed to look for inflammatory components, iron deposition, apoptosis, lipid deposition, and additional potential mechanisms underlying the increased collagen deposition. Our study only focused on the acute phase of CTRCD. Further studies are needed to rigorously evaluate the late occurrence of CTRCD in patients and the potential role of a comprehensive CMR exam that includes MRS in detecting late CTRCD.

## 7 Conclusion

Despite histological evidence of anthracycline-induced myocardial damage, there was considerable temporal variability and overlap in T_1_, T_2,_ and ECV within and between groups that did and did not have CTRCD, suggesting these CMR parameters may pose a challenge in detecting early potential CTRCD. Myocardial TG assessed by ^1^H-MRS at 12 weeks was markedly decreased in anthracycline-treated swine and was highly associated with the severity of histological myocardial damage, but these data should be considered preliminary and warrant further study.

## Funding

This study was supported by the grants from the 10.13039/100000050National Heart, Lung, and Blood Institute (NHLBI; R01HL127015) and 10.13039/100000968American Heart Association (AHA 15EIA22710040).

## Author contributions

**Jennifer Rodriguez:** Project administration, Conceptualization. **Tuyen Yankama:** Validation, Methodology, Formal analysis, Conceptualization. **Xiaoying Cai:** Writing – review and editing, Formal analysis, Conceptualization. **Selcuk Kucukseymen:** Writing – review and editing, Writing – original draft, Methodology, Investigation, Formal analysis, Data curation, Conceptualization. **Nadine Tung:** Investigation. **Kei Nakata:** Writing – original draft, Formal analysis, Conceptualization. **Reza Nezafat:** Writing – review and editing, Writing – original draft, Supervision, Resources, Investigation, Funding acquisition, Formal analysis, Conceptualization. **Warren J Manning:** Writing – review and editing, Conceptualization. **Shiro Nakamori:** Writing – review and editing, Writing – original draft, Formal analysis, Conceptualization. **Long Ngo:** Writing – original draft, Formal analysis, Conceptualization. **Patrick Pierce:** Conceptualization. **Eiryu Sai:** Writing – original draft, Formal analysis, Conceptualization.

## Ethics approval and consent

The study was approved by the Institutional Animal Care and Use Committee and conformed to the position of the American Heart Association on Research Animal Use and the Declaration of Helsinki.

## Consent for publication

Not applicable.

## Declaration of competing interests

Reza Nezafat, PhD, receives grant support from 10.13039/100000002NIH 5R01HL129185, 5R01HL127015, 1R01HL129157. The remaining authors have nothing to disclose.
